# Supplementary material for: Serum apolipoprotein A-I potentiates the therapeutic efficacy of lysocin E against Staphylococcus aureus
Source: Nat Commun. 2021 Nov 4;12:6364. doi: 10.1038/s41467-021-26702-0 (PMC8568920; doi:10.1038/s41467-021-26702-0)
Supplement: Supplementary file 1 — Supplementary Information [file 41467_2021_26702_MOESM1_ESM.pdf]

Supplementary information

Serum apolipoprotein A-I potentiates the therapeutic efficacy of lysocin E against *Staphylococcus aureus*

Hiroshi Hamamoto[1,2#], Suresh Panthee[3,#], Atmika Paudel[4], Kenichi Ishii[5], Jyunichiro Yasukawa[6], Su Jie[7], Atsushi Miyashita[1], Hiroaki Itoh[8], Kotaro Tokumoto[8], Masayuki Inoue[8], Kazuhisa Sekimizu[3,9]*

[1] Teikyo University Institute of Medical Mycology, Tokyo, Japan.

[2] Division of Sport and Health Science, Graduate School of Medical Care and Technology, Teikyo University, Tokyo, Japan.

[3] Drug Discoveries by Silkworm Models, Faculty of Pharma-Science, Teikyo University, Tokyo, Japan.

[4] International Institute for Zoonosis Control, Hokkaido University, Sapporo, Japan.

[5] Department of Biological Sciences, Graduate School of Science, The University of Tokyo, Tokyo, Japan.

[6] Department of Biochemistry, Faculty of Pharmaceutical Sciences, Doshisha Women's College of Liberal Arts, Kyoto, Japan.

[7] National Marine Environmental Monitoring Center, Dalian, China.

[8] Graduate School of Pharmaceutical Sciences, The University of Tokyo, Tokyo, Japan

[9] Genome Pharmaceuticals Institute, Ltd, Tokyo, Japan.

* e-mail: sekimizu@main.teikyo-u.ac.jp

[#] These authors contributed equally to this work

Supplementary information included in this file:

(i)	Supplementary Methods	2
(ii)	Supplementary Figures	7
(iii)	Supplementary Reference	27

Supplementary Methods

General Remarks. All reactions sensitive to air or moisture were performed under argon (Ar) atmosphere in dry solvents, unless otherwise noted. CH_2Cl_2 and DMF were purified by a Glass Contour Solvent Dispensing System (Nikko Hansen & Co., Osaka, Japan). All other reagents were used as supplied unless otherwise stated. Analytical thin-layer chromatography was performed using E. Merck Silica gel 60 F254 pre-coated plates. High performance liquid chromatography (HPLC) experiments were performed on an HPLC system equipped with a PU-4180 RHPLC pump (JASCO Products Co., Oklahoma City, OK, USA), an 1100 HPLC system (Agilent Technologies, Santa Clara, CA, USA), or a 1200 HPLC system (Agilent). ^{1}H and $^{13}C\{^{1}H\}$ nuclear magnetic resonance (NMR) spectra were recorded on an ECX 500 (500 MHz for ^{1}H NMR, 125 MHz for ^{13}C NMR) spectrometer (JEOL Ltd., Tokyo, Japan). Chemical shifts are denoted in δ (ppm) relative to residual solvent peaks as an internal standard ($CDCl_3$, ^{1}H δ 7.26, ^{13}C δ 77.0; DMSO-d_6, ^{1}H δ 2.50). Infrared spectra were recorded on an FT/IR-4100 spectrometer (JASCO). HRMS spectra were recorded on a MicrOTOFII (Bruker Daltonics, Billerica, MA, USA) electrospray ionisation time of flight (TOF) mass spectrometer. Optical rotations were recorded on a P-2200 polarimeter (JASCO).

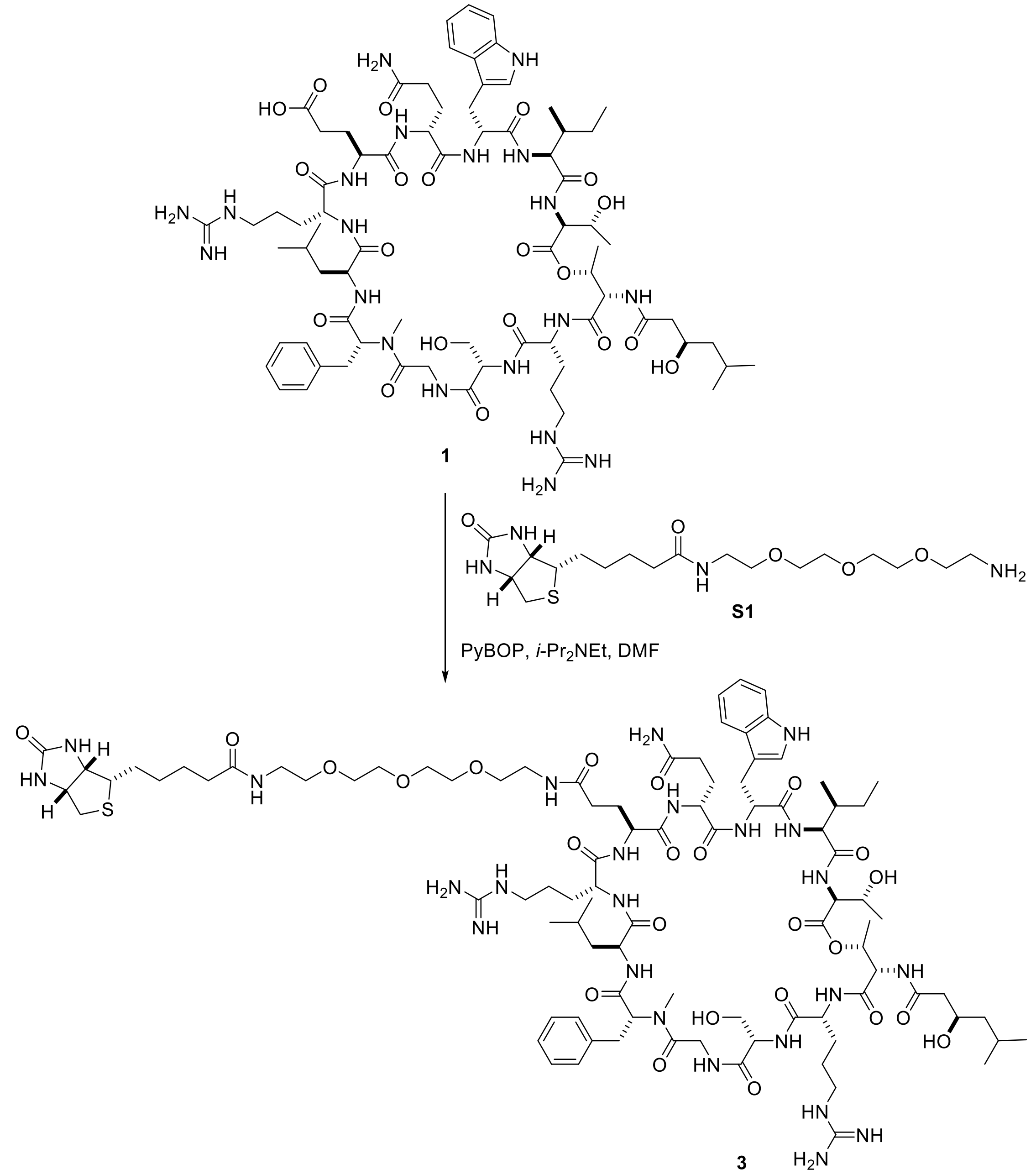

3 (Biotinylated lysocin E). A solution of PyBOP (3.12 mg, 6.00 μmol) in DMF (150 μL) and *i*-Pr_2NEt (1.95 μL, 12.0 μmol) was added to a solution of **1** (Lysocin E) (0.993 mg, 0.600 μmol) and amine **S1** (7.53 mg, 18.0 μmol) in DMF (150 μL) at 24 °C. The resultant mixture was stirred at 24 °C for 3 h. MeOH/H_2O (58.0/42.0) containing 0.05% TFA (400 μL) was added to the reaction mixture at 0 °C. The resultant solution was concentrated. The residue was purified by a first HPLC purification (column: Inertsil ODS-4 4.6 × 250 mm, eluent A: MeOH + 0.05% TFA, eluent B: H_2O + 0.05% TFA, A/B = 62.0/38.0, flow rate: 0.80 mL/min, temperature: 40 °C, detection: UV 280 nm), second HPLC purification (column: TSKgel Amide-80 7.8 × 300 mm, eluent A: MeCN + 0.05% TFA, eluent B: H_2O + 0.05% TFA, A/B = 88.0/12.0, flow rate: 2.0 mL/min, temperature: 40 °C, UV 280 nm), and third HPLC purification (column: Inertsil ODS-4 4.6 × 250 mm, eluent A: MeOH + 0.05% TFA, eluent B: H_2O + 0.05% TFA, linear gradient A/B = 53.0/47.0 to 78.0/22.0 over 50 min, flow rate: 2.0 mL/min, temperature: 40 °C, detection: UV 280 nm) to give

3 (0.583 mg, 0.258 μmol, 43%): white solid; HRMS (ESI-TOF) calcd for $C_{93}H_{150}N_{24}O_{24}S$ $[M+2H]^{2+}$ 1010.0499, found 1010.0511.

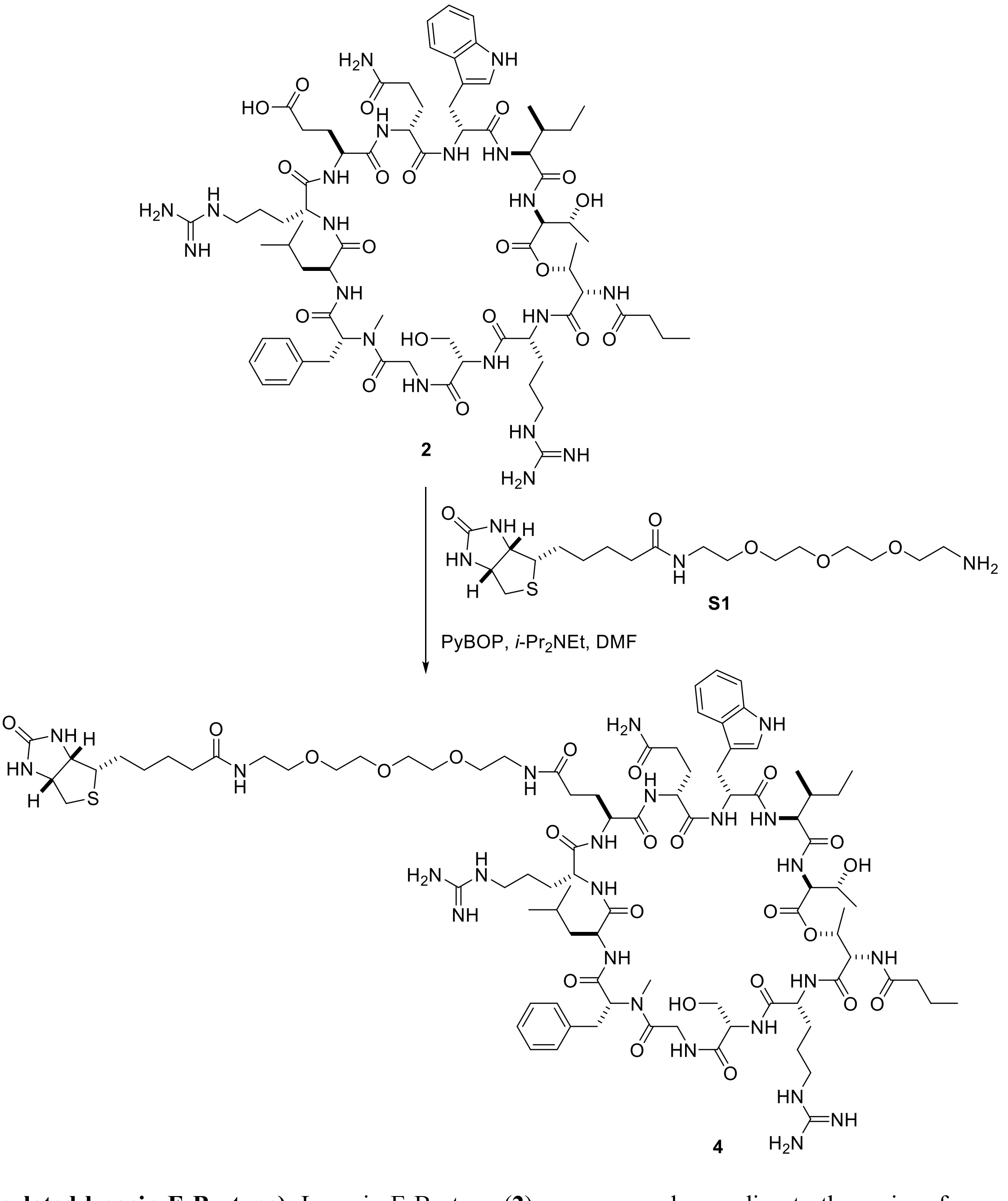

4 (Biotinylated lysocin E Bu-type). Lysocin E Bu-type (**2**) was prepared according to the main references[13].The HRMS and HPLC data of **2** were identical to those of the reported data [CAS 2027547-29-9].

A solution of PyBOP (3.73 mg, 7.16 μmol) in DMF (179 μL) and i-Pr_2NEt (2.32 μL, 14.3 μmol) was added to a solution of **2** (Lysocin E Bu-type) (1.20 mg, 0.716 μmol) and amine **S1** (9.00 mg, 21.5 μmol) in DMF (179 μL) at 29 °C. The resultant mixture was stirred at 29 °C for 3 h. H_2O containing 0.05% TFA (1.80 mL) was added to the reaction mixture at 0 °C. The resultant solution was lyophilised. The residue was dissolved in MeCN/H_2O (25.0/75.0) containing 0.05% TFA (5.00 mL). The solution was loaded onto an InertSep Slim C18-B column (360 mg). The

column was washed with MeCN/H_2O (25.0/75.0) containing 0.05% TFA (5.00 mL). The crude peptide was eluted with MeCN/H_2O (60.0/40.0) containing 0.05% TFA (10.0 mL). The eluate was lyophilised. The residue was purified by a first HPLC purification (column: TSKgel Amide-80 7.8 × 300 mm, eluent A: MeCN + 0.05% TFA, eluent B: H_2O + 0.05% TFA, A/B = 88.0/12.0, flow rate: 2.0 mL/min, temperature: 40 °C, detection: UV 280 nm) and second HPLC purification (column: Inertsil ODS-4 10 × 250 mm, eluent A: MeOH + 0.05% TFA, eluent B: H_2O + 0.05% TFA, A/B = 65.0/35.0, flow rate: 2.0 mL/min, temperature: 40 °C, detection: UV 280 nm) to give **4** (0.723 mg, 0.329 μmol, 46%): white solid; HRMS (ESI-TOF) calcd for $C_{90}H_{143}N_{24}O_{23}S$ $[M+H]^+$ 1960.0474, found 1960.0446.

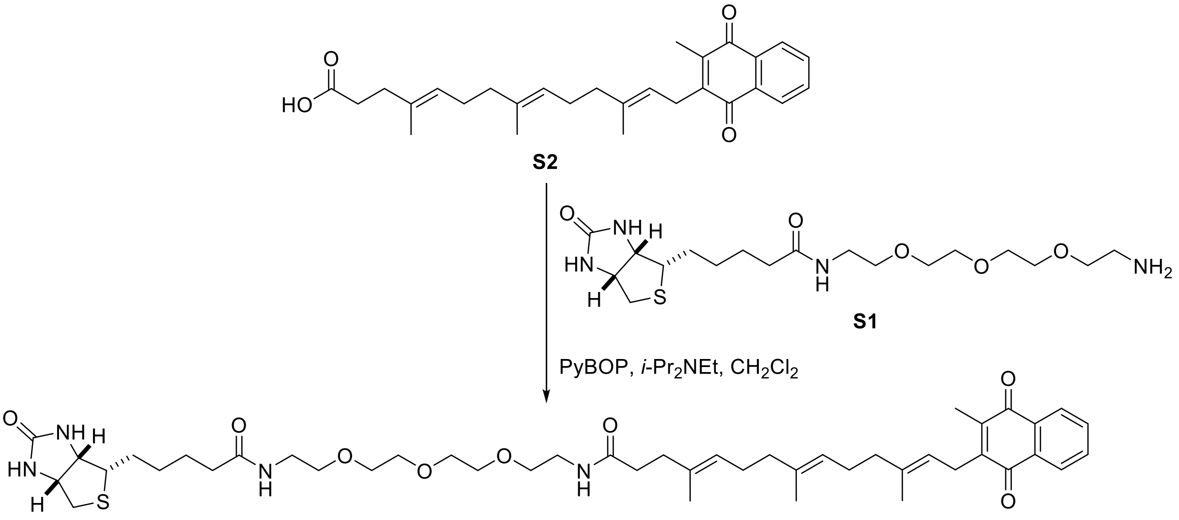

5 (Biotinylated menaquinone). Carboxylic acid **S2** was synthesised according to the literature[S1]. The ^{1}H NMR spectrum of **S2** was identical to that of the reported data [CAS 85216-29-1] [1].

A solution of PyBOP (6.57 mg, 12.6 μmol) in CH_2Cl_2 (56.2 μL) and a solution of *i*-Pr_2NEt (2.20 μL, 12.6 μmol) in CH_2Cl_2 (56.2 μL) were added to a solution of the above carboxylic acid **S2** (3.66 mg, 8.42 μmol) and amine **S1** (5.29 mg, 12.6 μmol) in CH_2Cl_2 (56.2 μL) at 0 °C. The reaction mixture was stirred at room temperature for 2 h, and then concentrated. The residue was purified by preparative thin-layer chromatography ($CHCl_3$/MeOH 10/1) to give biotinylated menaquinone **5** (5.14 mg, 6.15 μmol, 73%): yellow film; $[\alpha]_D^{23}$ +17.5 (*c* 0.257, $CHCl_3$); IR (film) 3287, 3081, 2922, 1696, 1655, 1549, 1451, 1293, 1100 cm^{-1}; ^{1}H NMR (500 MHz, $CDCl_3$) δ 1.41−2.29 (34H, m), 2.73 (1H, d, *J* = 12.6 Hz), 2.89 (1H, dd, *J* = 13.2, 5.2 Hz), 3.13 (1H, m), 3.35−3.62 (16H, m), 4.30 (1H, dd, *J* = 7.5, 4.6 Hz), 4.49 (1H, dd, *J* = 6.9, 5.2 Hz), 4.99−5.20 (3H, m), 5.51 (1H, s), 6.34 (1H, t, *J* = 5.2 Hz), 6.49 (1H, s), 6.71 (1H, t, *J* = 5.2 Hz), 7.68 (2H, dd, *J* = 5.7, 3.4 Hz), 8.06 (2H, m); $^{13}C\{^{1}H\}$ NMR (125 MHz, $CDCl_3$) δ 12.7, 15.9, 16.0, 16.4, 25.5, 26.0, 26.4, 26.7, 28.1, 35.3, 35.9, 39.1, 39.5, 39.6, 40.5, 55.5, 60.2, 61.7, 69.9−70.3 (8C), 119.1, 123.9, 124.9, 126.2, 126.3 (2C), 132.1, 133.29, 133.34 (2C), 133.7, 135.0, 137.4, 143.3, 146.1, 163.9, 173.1, 173.3, 184.5, 185.4; HRMS (ESI-TOF) calcd for $C_{46}H_{66}N_4O_8SNa$ $[M+Na]^+$ 857.4494, found 857.4472.

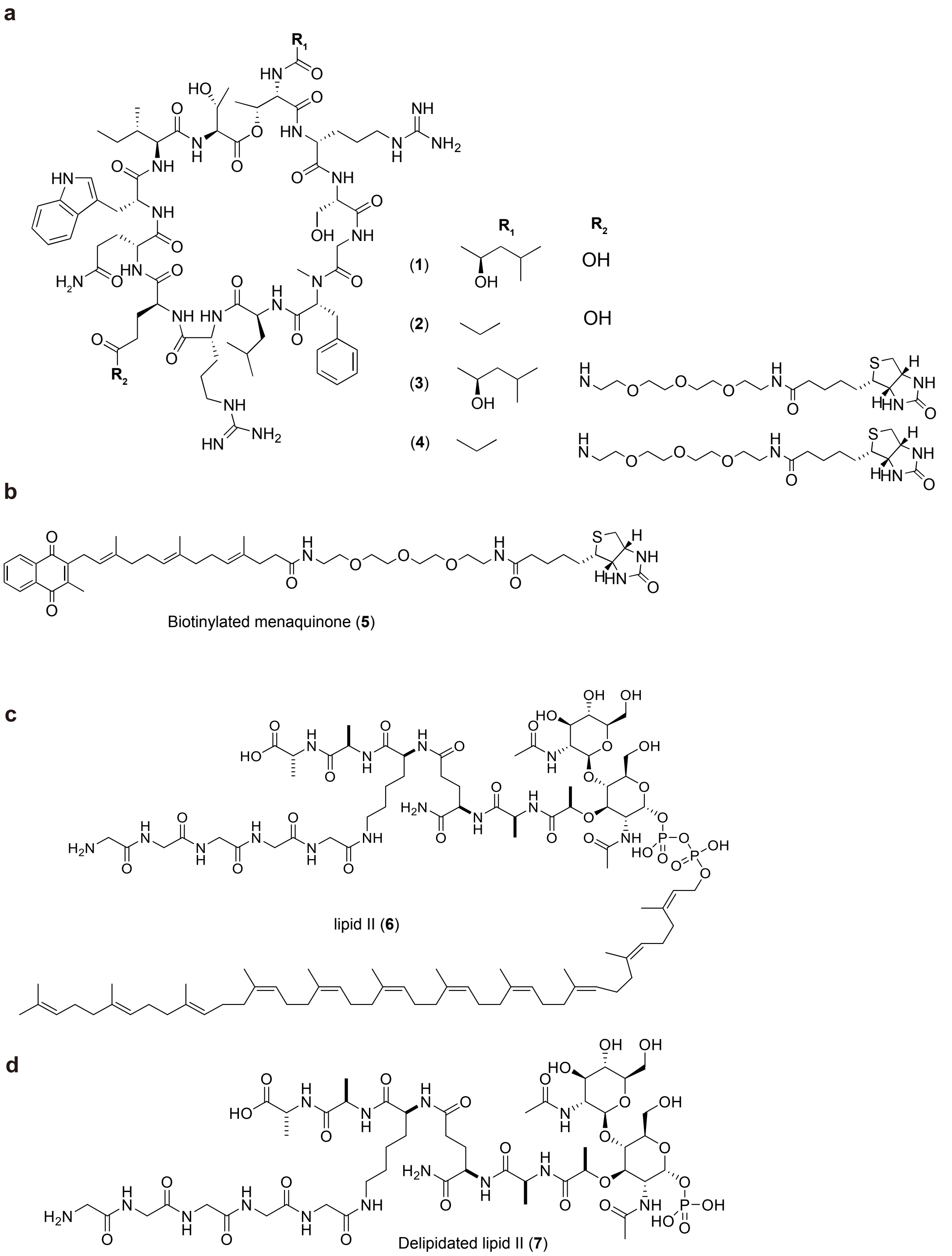

Supplementary Figure 1| Structure of compounds used in this study

a, Structure of lysocin E and lysocin E derivatives used in this study. Lysocin E natural type is **1**, Bu-type lysocin E is **2**, and biotinylated lysocin E natural-type and Bu-type used in biolayer interferometry (BLI) analysis or pull-down assay are **3** and **4**, respectively. **b**, Structure of biotinylated menaquinone (**5**) used in BLI analysis. **c, d**, Structure of *S. aureus* lipid II (**6)** and delipidated lipid II (**7**).

Supplementary Table 1| Effect of bovine serum on the antimicrobial activity of lysocin E against different microorganisms

Microorganisms / strains	MIC of lysocin E (μg ml^{-1})		Fold (A/B)
	Serum 0% (A)	Serum 10% (B)	
Staphylococcus aureus (MSSA)			
Smith ATCC13709	2	0.063	1/32
RN4220	4	0.13	1/32
Newman	4	0.13	1/32
MSSA1	4	0.13	1/32
S. aureus (MRSA)			
JE2 (USA300)	4	0.13	1/32
MRSA4	4	0.25	1/16
MR1 (Clinical isolate)	4	0.13	1/32
MR2 (Clinical isolate)	2	0.13	1/16
MR4 (Clinical isolate)	4	0.13	1/32
MR5 (Clinical isolate)	4	0.13	1/32
MR6 (Clinical isolate)	4	0.13	1/32
Staphylococcus haemolyticus JCM2416	2	0.0078	1/256
Staphylococcus pseudintermedius JCM17571	4	0.13	1/32
Bacillus subtilis JCM2499	2	0.25	1/8
Bacillus cereus JCM20037	2	0.50	1/4

Supplementary Table 2| rhApoA-I enhanced antimicrobial activity of lysocin E against various MSSA and MRSA strains

	Strains	MHB (control)	+rhApoA-I 25µg ml^{-1}
MSSA	ATCC13709 (Smith)	2	0.25
	RN4220	4	0.5
	Newman	4	0.5
	MSSA1	4	0.5
MRSA	JE2 (USA300)	4	0.5
	MRSA4 (Clinical isolate)	4	0.13
	MR1 (Clinical isolate)	4	0.25
	MR2 (Clinical isolate)	2	0.25
	MR4 (Clinical isolate)	4	0.5
	MR5 (Clinical isolate)	4	0.25
	MR6 (Clinical isolate)	4	0.25

a

Fraction	Protein (mg)	Total activity (unit)	Yield (%)	Specific activity (unit mg^{-1} protein)	Purification (fold)
I. Calf serum	1100	13000	100	11	1
II. Ethanol extract	17	20000	160	1200	110
III. ODS column	4.3	15000	120	3600	330
IV. Superose12 column (peak 1)	3.4	20000	160	5900	540
IV. Superose12 column (peak 2)	0.2	1400	11	7000	640

b

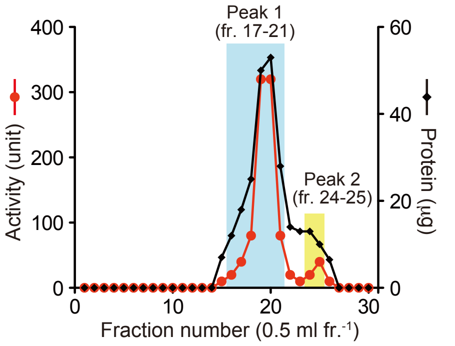

c

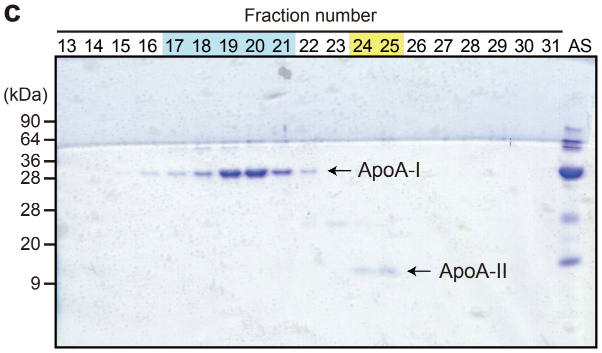

d

Peak 1; Apolipoprotein A-I [*Bos taurus*]

```
1   RHFWQQDDPQSSWDRVKDFATVYVEAIKDSGRDYVAQFEA  40
41  SALGKQLNLKLLDNWDTLASTLSKVREQLGPVTQEFWDNL  80
81  EKETASLRQEMHKDLEEVKQKVQPYLDEFQKKWHEEVEIY 120
121 RQKVAPLGEEFREGARQKVQELQDKLSPLAQELRDRARAH 160
161 VETLRQQLAPYSDDLRQRLTARLEALKEGGGSLAEYHAKA 200
201 SEQLKALGEKAKPVLEDLRQGLLPVLESLKVSILAAIDEA 240
241 SKKLNAQ                                  247
```

Peak 2; Apolipoprotein A-II [*Bos taurus*]

```
1   ALVRRQAEESNLQSLVSQYFQTVADYGKDLVEKAKGSELQ  40
41  TQAKAYFEKTQEELTPFFKKAGTDLLNFLSSFIDPKKQPA  80
81  TR
```

e

ApoA species	Specific activity (unit mg^{-1} protein)
rmApoA-I	6400
rhApoA-I	6500
hApoA-II	5900

f

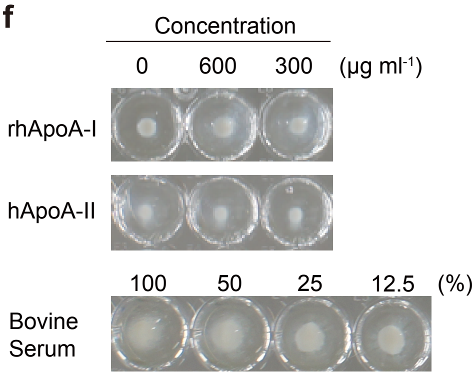

g

Concentration (µg mL^{-1})		MIC (µg mL^{-1})
rhApoA-I	hApoA-II	
-	-	4
32	0	0.5
0	6.3	1
32	6.3	0.5

Supplementary Figure 2| Biochemical identification of apolipoprotein A-I as an enhancing factor of lysocin E activity

a, Purification table of lysocin E antimicrobial enhancing factors from bovine serum. **b**, Elution patterns of the Superose12 column chromatography. The black line shows the amount of protein (right axis), and the red line shows the lysocin E antimicrobial promoting activity (left axis) in each fraction. AS, applied sample. **c**, Coomassie Brilliant Blue-stained images after separation by 12% SDS-PAGE for fractions 13 to 31 obtained from the Superose12 column chromatography. **d**, Amino acid sequence identified by peptide mass fingerprinting analysis for samples eluted from SDS-PAGE gels. Red letters indicate fragments obtained by peptide mass fingerprinting analysis. **e**, Specific activities of mouse and rhApoA-I, and purified hApoA-II for lysocin E antimicrobial enhancing activity. **f**, ApoA-I, II and bovine serum did not show antimicrobial activity against *S. aureus* after overnight incubation. **g**, Effect of mixing human ApoA-I and II against the MIC of lysocin E.

a

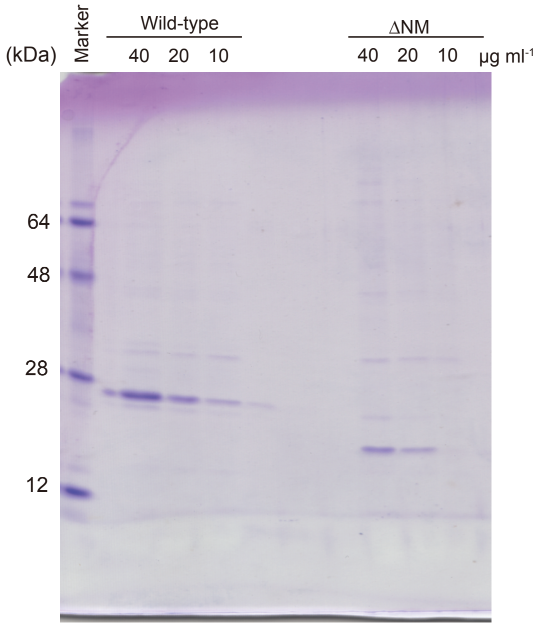

b

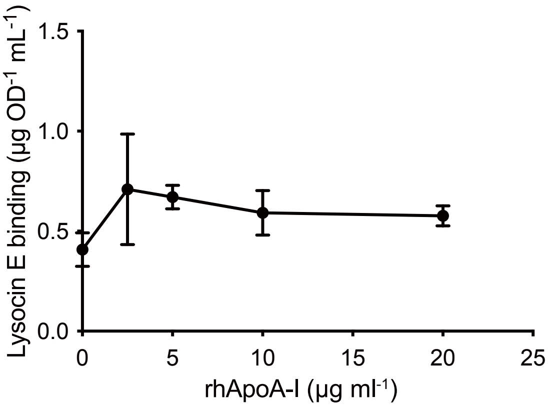

Supplementary Figure 3| Binding of rhApoA-I to the cell surface of *S. aureus* and effect on lysocin E binding
a, Representative SDS-PAGE image for calculating of the amount of rhApoA-I and rhApoA-I ΔNM bound to the cell surface of *S. aureus*. **b**, Effect of rhApoA-I on the amount of lysocin E that binds to the cell surface of *S. aureus*. RN4220 (2 ml, OD_{600} = 0.5) was incubated for 5 min with the indicated concentrations of rhApoA-I in MHB, and further incubated with 2 μg ml^{-1} lysocin E. The cells were precipitated by centrifugation at 6000 x *g* for 5 min and washed with the same volume of PBS (-). Lysocin E was extracted from precipitated cells by 50% acetone and analysed by reversed phase-HPLC. HPLC was performed as previously described[9]. Data represent mean ± SEM of triplicate results.

Supplementary Table 3| Antimicrobial activity of derivatives of lysocin E and nisin against *S. aureus* RN4220 strain in the presence of rhApoA-I

Compounds	MIC (μg ml^{-1})	
	Control	+rhApoA-I 25μg ml^{-1} (fold)
Lysocin E (natural, **1**)	4	0.5 (1/8)
Bu type lysocin E (**2**)	4	4 (1/1)
Nisin	8	2 (1/4)
Vancomycin	1	1 (1/1)
Daptomycin	0.5	0.5 (1/1)
Lysocin E (1) with lipid II 25 μg ml^{-1}	4	4 (1/1)
Nisin with lipid II 25 μg ml^{-1}	>32	>32

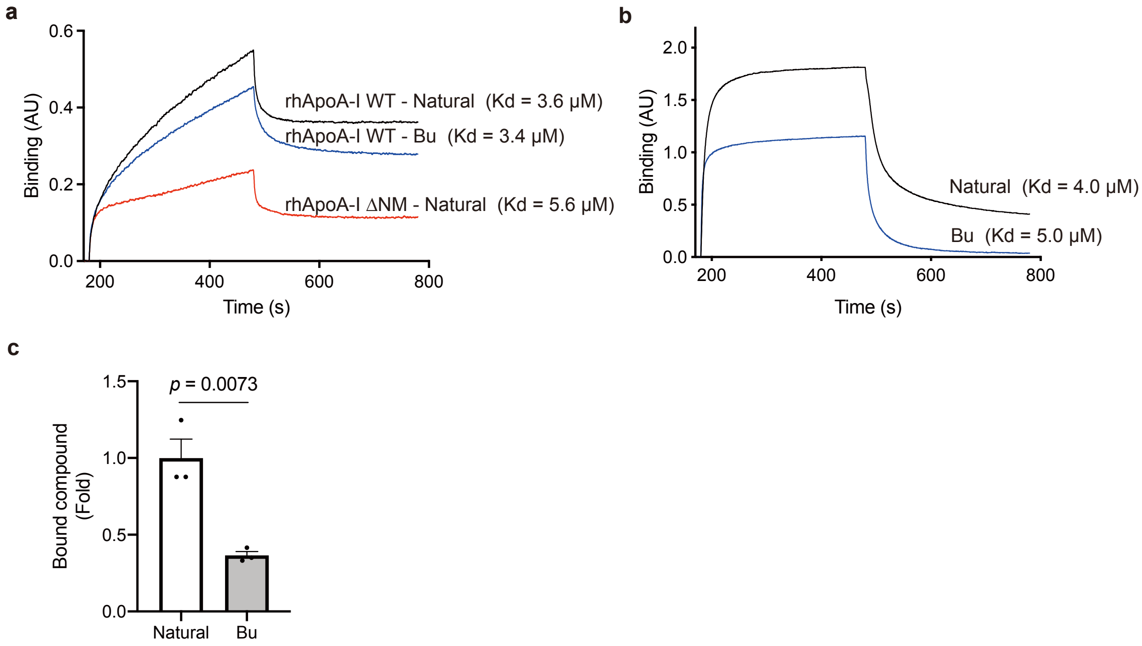

Supplementary Figure 4| Comparison of natural- and Bu-type lysocin E with regard to their interactions with ApoA-I, menaquinone, and lipid II

a, BLI analysis of the rhApoA-I (wild type or ΔNM variant) and natural- or Bu-type lysocin E interaction. Biotinylated natural or Bu type lysocin E (**3**, **4**, Supplementary Fig. 1a) was immobilised on a streptavidin biosensor and kinetic analysis was performed using 2.5 µM protein. **b**, BLI analysis for menaquinone and natural or Bu-type lysocin E interaction. Biotinylated menaquinone (**5**, Supplementary Fig. 1b) was immobilised on a streptavidin biosensor and kinetic analysis was performed using 12.5 µM lysocin E derivatives. Representative data showing similar results from 3 independent experiments are shown (**a, b**). **c**, Lipid II binding to biotinylated natural- or Bu-type lysocin E in a pull-down assay. Data represent mean ± SEM from 3 independent experiments, and statistical analysis was performed using the unpaired Student 2-tailed *t*-test.

a

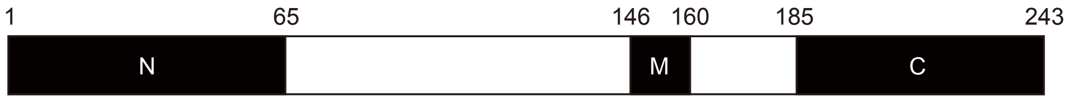

b

rhApoA-I	Relative activity (%)
WT	100
∆N	38
∆M	58
∆C	100
∆NM	<11
∆NC	38

Supplementary Figure 5| rhApoA-I variant used in this study.
a, The deletion region of rhApoA-I. **b**, The relative lysocin E antimicrobial-enhancing activity of the rhApoA-I variant.

a

```
  1 gacgacccgc cgcagagccc atgggatcgc gtgaaggacc tggccactgt gtacgtggat
 61 gtgctgaaag acagcggccg cgactatgtg tcccagtttg aaggctccgc cctgggcaaa
121 caactgaacc tgaagctgct ggacaactgg gacagcgtga cgtccacctt cagcaagctg
181 cgcgaacagc tgggcccggt gacccaggaa ttctgggata acctggaaaa ggagaccgag
241 ggcctgcgcc aggagatgag caaggatctg gaggaggtga aggccaaggt gcagccgtac
301 ctggacgact tccagaagaa gtggcaggag gagatggagc tgtaccgcca gaaggtggag
361 ccgctgcgcg cagagctgca ggagggcgcg cgccagaagc tgcacgagct gcaagagaag
421 ctgagcccac tgggcgagga gatgcgcgac cgcgcgcgcg cccatgtcga cgcgctgcgc
481 acgcatctgg cgccgtacag cgacgagctg cgccagcgcc tggcggcgcg cctggaggct
541 ctgaaggaga acggcggcgc ccgcctggcc gagtaccacg ccaaggccac cgagcatctg
601 agcacgctga gcgagaaggc caagccggcg ctggaggacc tgcgccaagg cctgctgccg
661 gtgctggaga gcttcaaggt cagcttcctg agcgctctgg aagagtacac taagaagctg
721 aacacccagt ga
```

b

```
  1 gatgaaccgc agagtcagtg ggataaagtg aaagattttg cgaacgtgta tgtggatgcg
 61 gtgaaagata gcggccgcga ttatgtgagt cagtttgaaa gcagcagcct gggtcagcag
121 ctgaacctga acctgctgga aaactgggat accctgggca gcaccgtgag tcagctgcaa
181 gaacgcctgg gcccgctgac ccgcgatttt tgggataacc tggaaaaaga aaccgattgg
241 gtgcgccaag aaatgaacaa agatctggaa gaagtgaaac agaaagtgca gccgtatctg
301 gatgaatttc agaaaaaatg gaaagaagat gtggaactgt atcgtcagaa agtggcgccg
361 ctgggcgcgg aactgcaaga aagcgcgcgt cagaaactgc aagaactgca aggccgcctg
421 agcccggtgg cggaagaatt tcgcgatcgc atgcgcaccc atgtggatag cctgcgcacg
481 cagctggcgc cgcatagcga acagatgcgc gaaagcctgg cgcagcgcct ggcggaactg
541 aaaagcaacc cgaccctgaa cgaatatcat acacgcgcga aaacccatct gaaaaccctg
601 ggcgaaaagg cgcgcccggc gctggaagat ctgcgccata gcctgatgcc gatgctggaa
661 accctgaaaa cccaagtgca gagcgtgatt gataaagcga gcgaaaccct gaccgcgcag
721 taa
```

Supplementary Figure 6| Optimised sequences for recombinant ApoA-I
The sequence for production of recombinant ApoA-I of human (a) and mouse (b).

Supplementary Table 4| Nucleotide sequences of primers used in this study.
Letters in lower case indicate linker sequences and underlines indicate restriction enzyme recognition sites.

Target	Forward	Reverse
rhApoA-I ΔN, nucleotide No. 196-729	5'-GGTACCCATATGCATCACCATCACCATCACCCGGTGACCCAGGAATTCT-3' (ΔN F)	5'-GGTACCAAGCTTAGATCTACTAGTTCTAGATCACTGGG-3' (ΔN R)
rhApoA-I ΔM, nucleotide No. 1-435	5'-GGTACCCATATGCATCACCATC-3' (Apo F)	5'-gcccagtgggctcagcttctctt-3'
rhApoA-I ΔM, nucleotide No. 481-729	5'-agaagctgagcccactgggcACGCATCTGGCGCCGTACAG-3'	5'-AAGCTTAGATCTACTAGTTCTAGATCACTGGG-3'
ΔC	Apo F	5'- GGTACCAAGCTTTCAGTTCTCCTTCAGAGCCTCCA-3' (ΔC R)

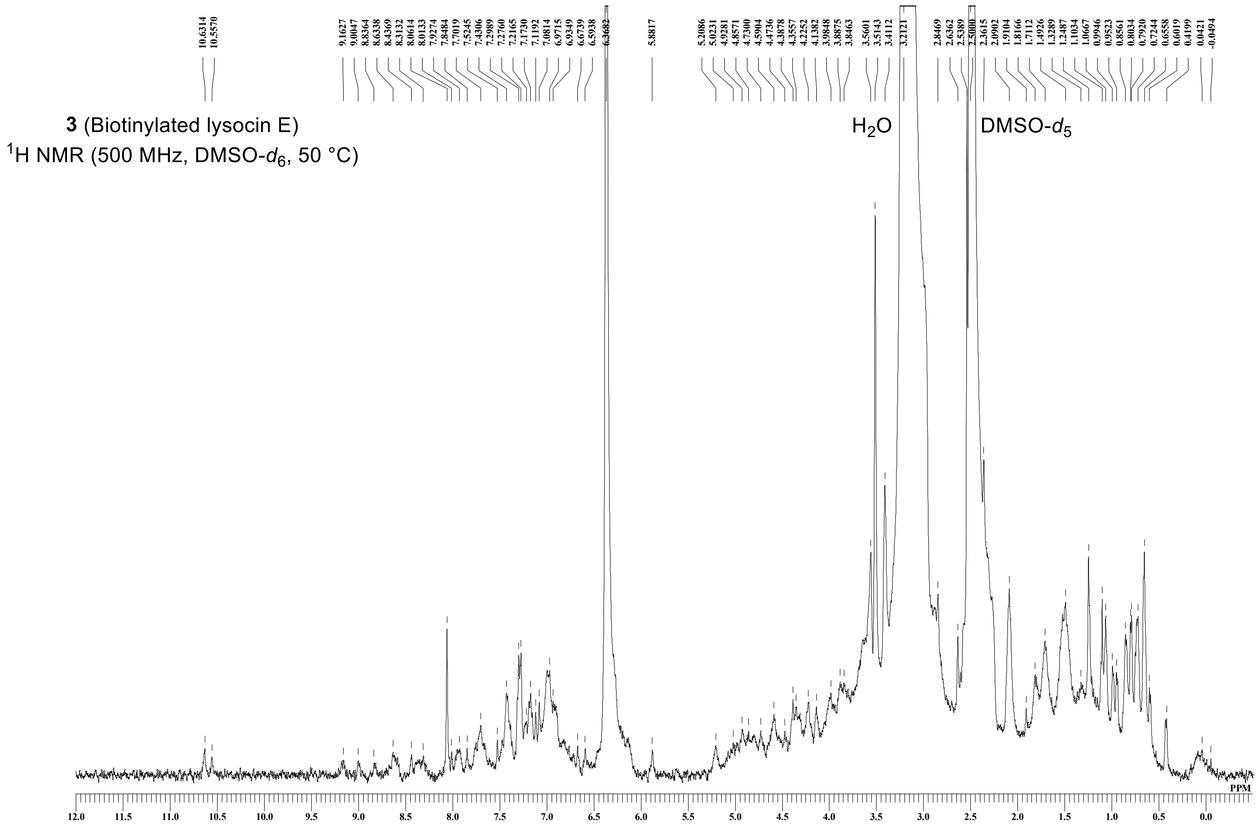

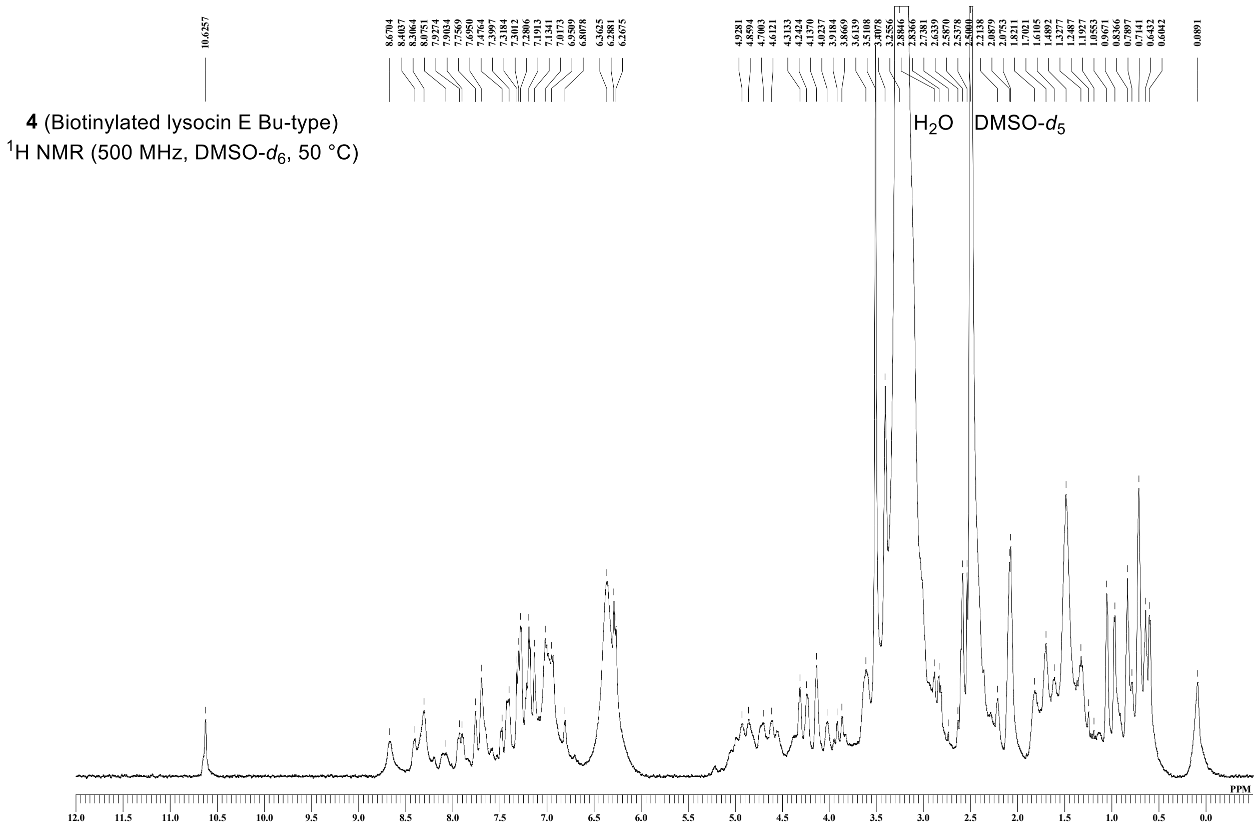

Supplementary Figure 7| ^{1}H NMR spectra of **3** and **4**.

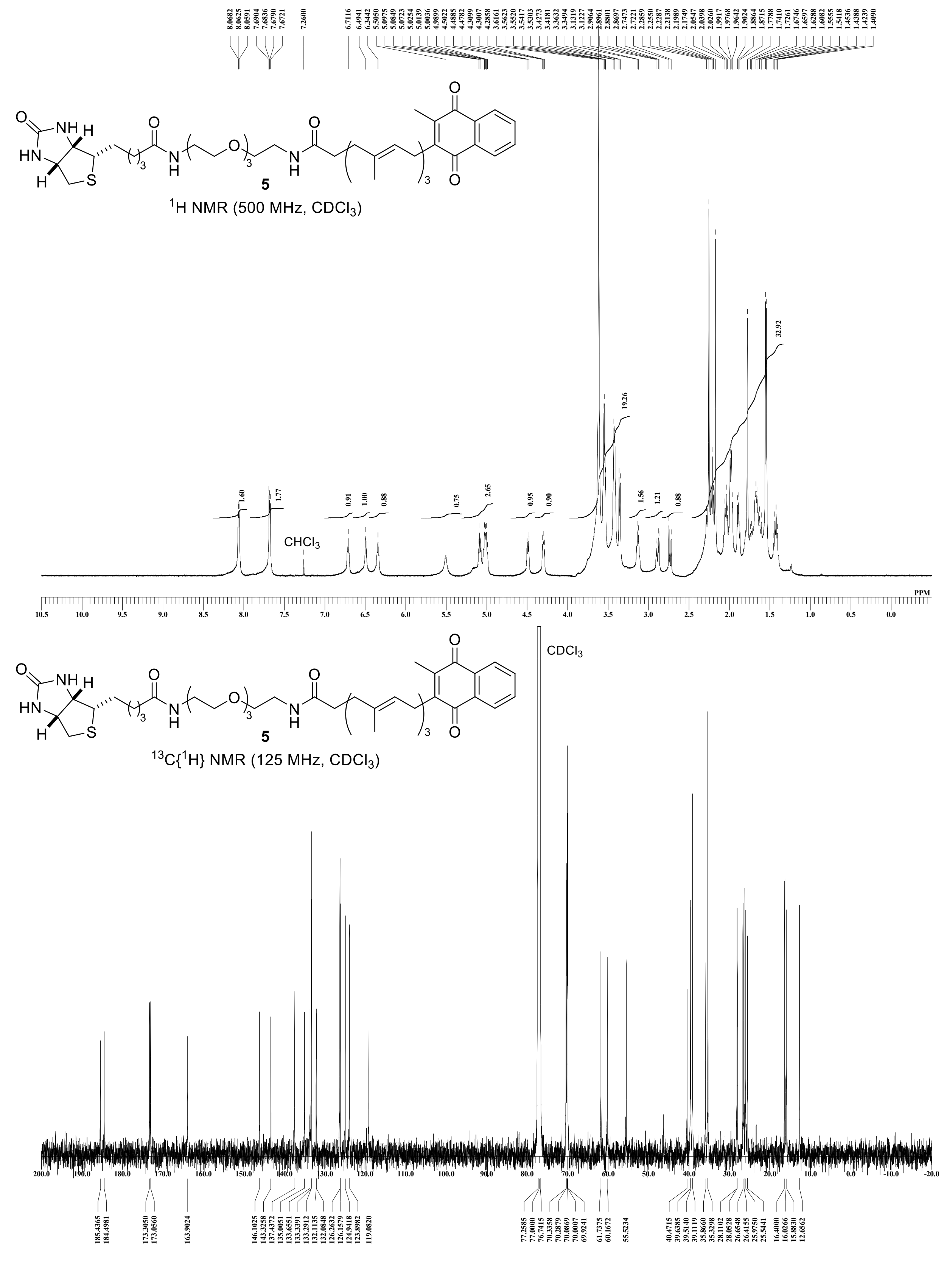

Supplementary Figure 8| ^{1}H and ^{13}C{^{1}H} NMR spectra of 5 (Biotinylated menaquinone)

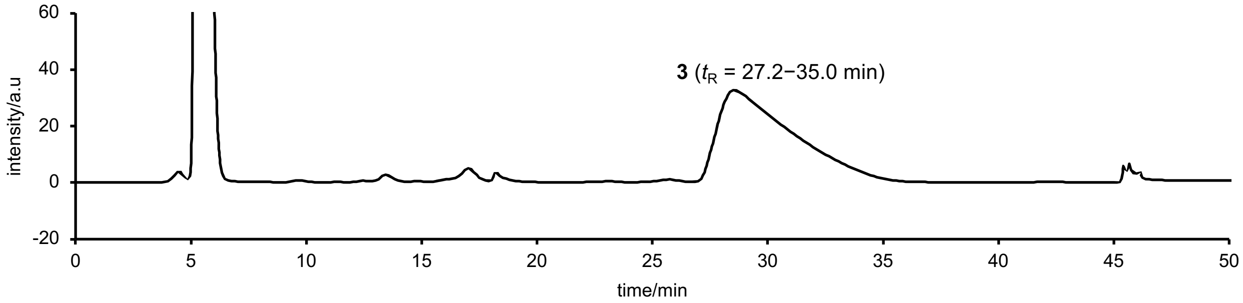

Supplementary Figure 9| HPLC chromatogram for first HPLC purification of 3 (Biotinylated lysocin E)
Column: Inertsil ODS-4 4.6 × 250 mm, eluent A: MeOH + 0.05% TFA, eluent B: H_2O + 0.05% TFA, A/B = 62.0/38.0, flow rate: 0.80 mL/min, temperature: 40 °C, detection: UV 280 nm.

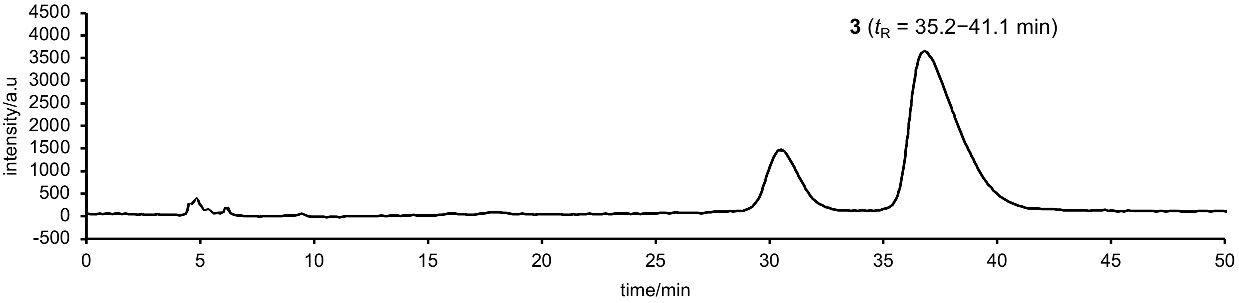

Supplementary Figure 10| HPLC chromatogram for second HPLC purification of 3 (Biotinylated lysocin E) Column: TSKgel Amide-80 7.8 × 300 mm, eluent A: MeCN + 0.05% TFA, eluent B: H_2O + 0.05% TFA, A/B = 88.0/12.0, flow rate: 2.0 mL/min, temperature: 40 °C, UV 280 nm.

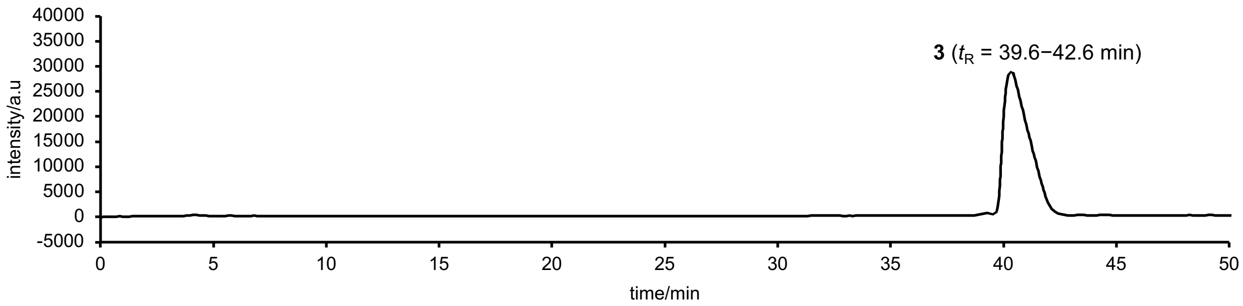

Supplementary Figure 11| HPLC chromatogram for third HPLC purification of 3 (Biotinylated lysocin E)
Column: Inertsil ODS-4 4.6 × 250 mm, eluent A: MeOH + 0.05% TFA, eluent B: H_2O + 0.05% TFA, linear gradient A/B = 53.0/47.0 to 78.0/22.0 over 50 min, flow rate: 2.0 mL/min, temperature: 40 °C, detection: UV 280 nm.

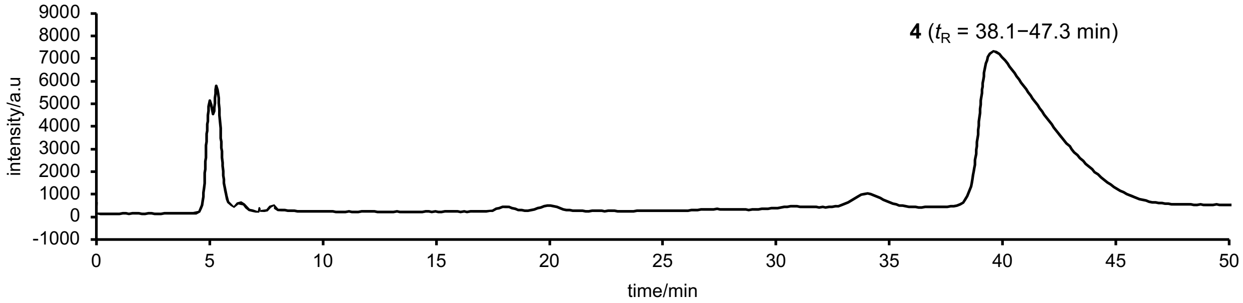

Supplementary Figure 12| HPLC chromatogram for first HPLC purification of 4 (Biotinylated lysocin E Bu-type)
Column: TSKgel Amide-80 7.8 × 300 mm, eluent A: MeCN + 0.05% TFA, eluent B: H_2O + 0.05% TFA, A/B = 88.0/12.0, flow rate: 2.0 mL/min, temperature: 40 °C, UV 280 nm.

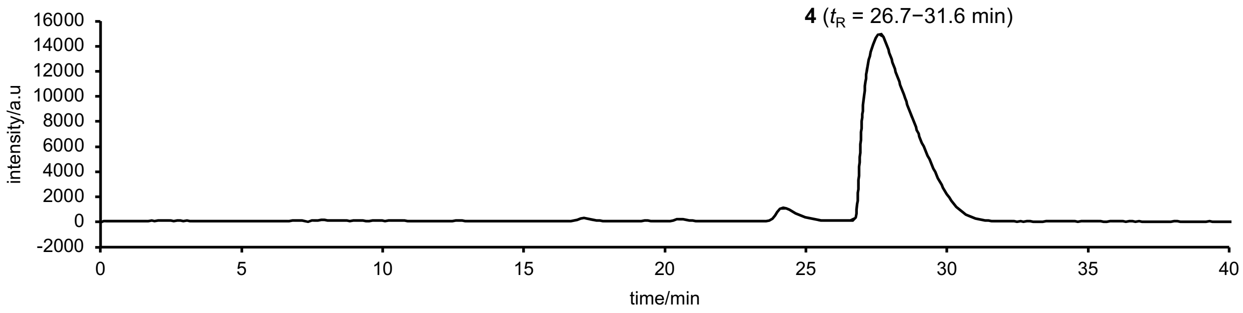

Supplementary Figure 13| HPLC chromatogram for second HPLC purification of 4 (Biotinylated lysocin E Bu-type)

Column: Inertsil ODS-4 10 × 250 mm, eluent A: MeOH + 0.05% TFA, eluent B: H_2O + 0.05% TFA, A/B = 65.0/35.0, flow rate: 2.0 mL/min, temperature: 40 °C, detection: UV 280 nm.

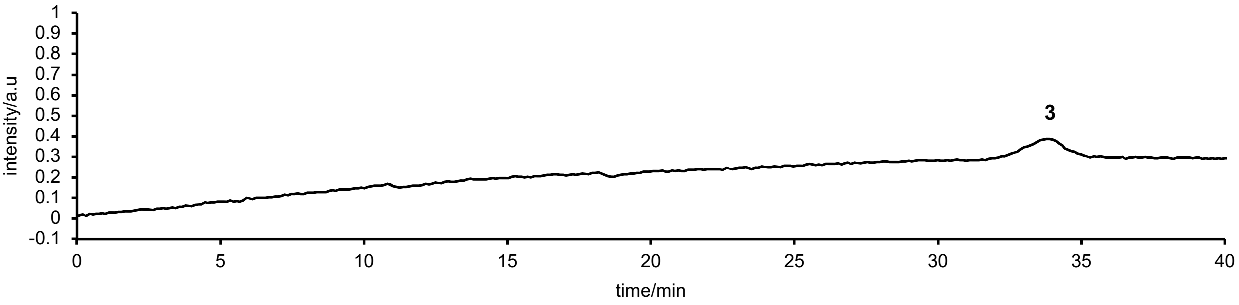

Supplementary Figure 14| HPLC chromatogram of purified 3 (Biotinylated lysocin E)
Column: Inertsil ODS-4 4.6 × 250 mm, eluent A: MeOH + 0.05% TFA, eluent B: H_2O + 0.05% TFA, A/B = 64.0/36.0, flow rate: 0.50 mL/min, temperature: 40 °C, detection: UV 280 nm.

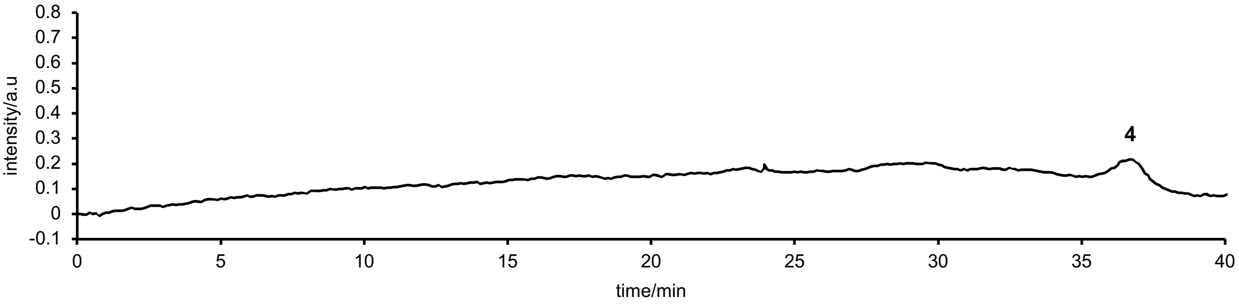

Supplementary Figure 15| HPLC chromatogram hart of purified 4 (Biotinylated lysocin E Bu-type).
Column: Inertsil ODS-4 4.6 × 250 mm, eluent A: MeOH + 0.05% TFA, eluent B: H_2O + 0.05% TFA, A/B = 62.0/38.0, flow rate: 0.50 mL/min, temperature: 40 °C, detection: UV 280 nm.

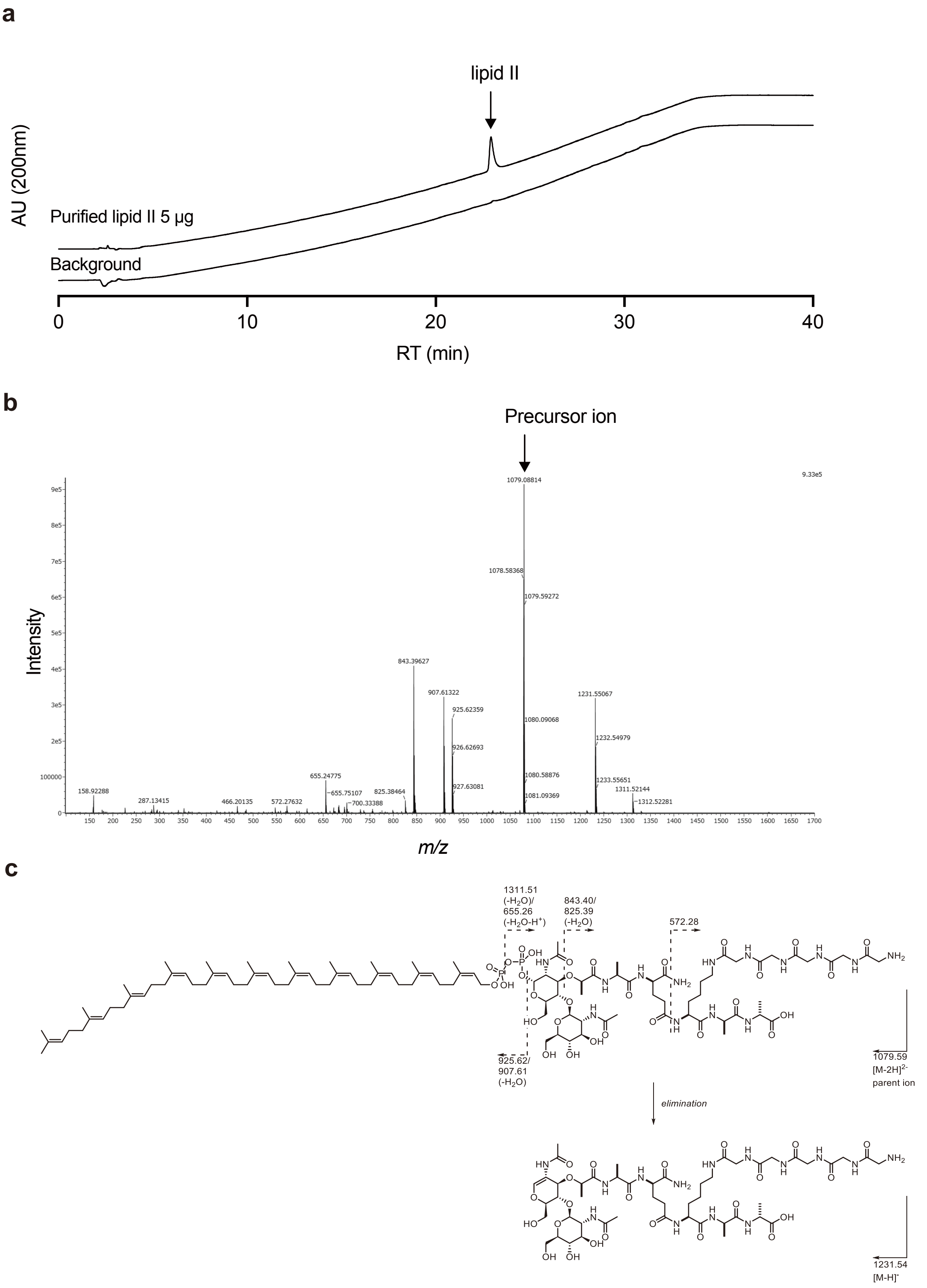

Supplementary Figure 16| Purity and structural confirmation of lipid II

a, HPLC chromatograph of purified lipid II. **b**, MS/MS spectrum of purified lipid II. **c**, Assignment of characteristic fragmentation ions.

Supplementary Reference

1 Fujii, S. *et al*. Systematic synthesis and anti-inflammatory activity of omega-carboxylated menaquinone derivatives--Investigations on identified and putative vitamin K(2) metabolites. *Bioorg. Med. Chem.* **23**, 2344-2352, doi:10.1016/j.bmc.2015.03.070 (2015).
